# Diagnosis of Froin’s Syndrome by Parallel Analysis of Ventriculoperitoneal Shunt and Lumbar Cerebrospinal Fluid in a Patient with Cervical Spinal Stenosis

**DOI:** 10.3390/jcm12155012

**Published:** 2023-07-30

**Authors:** Franca Laura Fries, Benedict Kleiser, Patricia Schwarz, Maria P. Tieck, Kornelia Laichinger, Annerose Mengel, Ulf Ziemann, Markus C. Kowarik

**Affiliations:** 1 Department of Neurodegeneration, Center of Neurology, Hertie-Institute for Clinical Brain Research, University of Tuebingen, 72076 Tuebingen, Germany; 2 German Center for Neurodegenerative Diseases, University of Tuebingen, 72076 Tuebingen, Germany; 3 Department of Epileptology, Hertie-Institute for Clinical Brain Research, University of Tuebingen, 72076 Tuebingen, Germany; 4 Department of Neurology and Stroke, Hertie-Institute for Clinical Brain Research, University of Tuebingen, 72076 Tubingen, Germany; patricia.schwarz@med.uni-tuebingen.de (P.S.); maria.tieck-fernandez@med.uni-tuebingen.de (M.P.T.); kornelia.laichinger@med.uni-tuebingen.de (K.L.); annerose.mengel@med.uni-tuebingen.de (A.M.); ulf.ziemann@med.uni-tuebingen.de (U.Z.); markus.kowarik@med.uni-tuebingen.de (M.C.K.)

**Keywords:** Froin’s syndrome, cervical spinal stenosis, ventriculoperitoneal shunt, lumbar puncture, elevated CSF protein

## Abstract

Elevated protein levels in cerebrospinal fluid (CSF) can occur in various pathologies and are sometimes difficult to interpret. We report a 62-year-old male patient with subacute neurological deterioration, progressive tetraparesis, and cytoalbumin dissociation in the lumbar CSF. The patient had a pre-existing cervical spinal stenosis with mild tetraparesis. Based on the initial cytoalbumin dissociation (protein 938 mg/dL, 4 leucocytes/µL), Guillain–Barré syndrome was initially considered. For further diagnosis, a CSF sample was taken from a pre-existing ventriculoperitoneal shunt, which showed a normal protein and cell count considering the patient’s age (protein 70 mg/dL, 1 leucocyte/µL). In conclusion, we suggest that intermediate aggravation of tetraparesis was due to pneumonia with septic constellation, and the cytoalbumin dissociation was interpreted as Froin’s syndrome (FS) due to spinal stenosis. In this unique case, we were able to prove the -often suspected- case of FS by parallel analysis of ventriculoperitoneal shunt and lumbar CSF. The triad of xanthochromia, high protein levels, and marked coagulation was first described by Georges Froin and occurs in various processes leading to severe spinal stenosis. The altered composition of lumbar CSF might be due to impaired CSF circulation; however, the exact mechanisms of this phenomenon require further investigation.

## 1. Background

Cerebrospinal fluid (CSF) analysis is an important diagnostic tool in the workup of neurological diseases. An elevation of CSF protein level may indicate intrathecal protein synthesis or may occur in the context of a disrupted blood–brain barrier (BBB) during infections, leptomeningeal metastasis, or subarachnoid hemorrhage. As an alternative cause for increased CSF protein levels, disturbances in CSF circulation between the upper and lower spinal cord can falsify the CSF results from lumbar punctures. Highly elevated CSF protein levels in lumbar puncture should lead to the differential diagnosis of Froin’s syndrome (FS) during routine clinical practice [1]. Although FS has been recognized since 1903, the exact cause of the protein increase in spinal stenosis is not fully understood yet. In the following case, we clearly confirmed FS by parallel analysis of CSF from a ventriculoperitoneal shunt and a lumbar puncture in a multimorbid elderly patient and were thus able to exclude differential diagnoses such as Guillain–Barre syndrome.

## 2. Case Presentation

**Figure 1:**
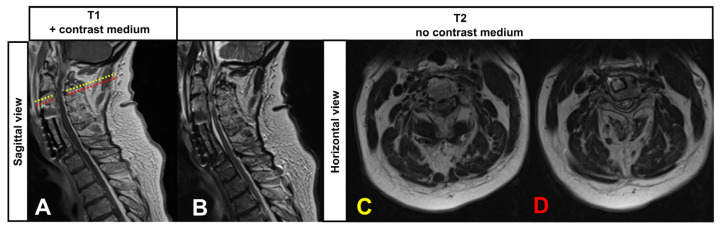
Cervical spinal stenosis and surgical treatment in the ventral area of cervical vertebrae 3/4. The cervical stenosis (Kang grade 3) and myelopathy were stable compared to previous MRIs. (**A**,**B**) Sagittal view. Levels of the horizontal slides (**C**,**D**) are indicated with dotted lines: in yellow is the level of the stenosis (**C**), and in red is the level of the myelomalacia directly caudal to the stenosis (**D**).

We here report on a 62-year-old male patient who presented with increasing subacute neurological deterioration of a known tetra-paresis and impoverishment of speech production within one week. A cervical spinal canal stenosis (multi-segmental with consecutive myelomalacia and attempt at surgical care, Figure 1A–D), as well as right frontobasal parenchymal defects following subarachnoid hemorrhage with consecutive ventricular congestion, ventriculoperitoneal shunt insertion in 2006 and pituitary insufficiency, were known from the previous medical history. Shortly before admission, the patient was wheelchair-mobile, could walk with support, and had speech production described as largely normal and adequate. There were no traumatic events that could explain an increase in tetraparesis.

Initially, the patient was admitted to intensive care because of a progressive respiratory deterioration due to pneumonia with septic constellation, including hypotension and tachycardia and without any explanatory pathogen in the tracheal secretions and blood cultures, which was treated with antibiotics (piperacillin/tazobactam and clarithromycin) and supportive O_2_ high-flow therapy.

After pulmonary stabilization, the patient was transferred to the neurological immediate care (IMC) for further diagnostics and treatment of the progressive tetra-paresis and vigilance mitigation.

On admission, the clinical–neurological examination revealed a somnolent patient in reduced general condition, oriented to person only, without meningism. Cranial nerves were unremarkable, and no sensory disturbances were notable. A pronounced tetra-paresis with an emphasis on the left side and especially lower extremities, as well as generalized areflexia, were detectable. Disturbances of bladder and bowel function were not evident.

Cranial imaging with computer tomography and magnetic resonance imaging (MRI) showed no evidence of acute ischaemia, hemorrhage, or elevated intracranial pressure. Shunt dysfunction was excluded by careful assessment of ventricular size, shunt location, and puncture by the neuroradiologists and neurosurgeons. We also compared CSF parameters of the ventriculoperitoneal shunt before the actual clinical deterioration from a previous clinical stay and did not observe significantly different parameters compared to the current analysis, also indicating stable shunt function and location. The pre-existing right frontal parenchymal defect and pituitary adenoma were unchanged from previous imaging.

An additional MRI of the spinal cord showed myelopathy in the T2-weighted images at the level of cervical vertebra 3 and a narrowed spinal canal (Kang grade 3) constant to the previous findings 5 years ago (see Figure 1A–D) and showed no signs of recent alterations including trauma-related changes. Laboratory findings on admission showed an elevated CRP, pancytopenia, leukocytopenia, and thrombocytopenia due to sepsis. The hormone axes were largely normal on day 3 in the context of pituitary insufficiency. A lumbar puncture at the level of lumbar vertebrae 3 and 4 to exclude cerebral infection revealed a marked cytoalbumin dissociation (protein 938 mg/dL, albumin 7670 mg/L, 4 leucocytes/µL, lactate slightly increased with 2.5 mmol/L, glucose regular 50 mg/dL, xanthochrome color of the CSF, details see Table 1). IL6 in the CSF was slightly increased by 52 ng/L.

In the context of clinically worsened tetraparesis and cytoalbumin dissociation in the CSF, the diagnosis of Guillain–Barré syndrome was first considered, and intravenous immunoglobulins started. Additional neurographies showed conspicuous F-waves and mild amplitude reductions, which had already been described in a previous neurological work-up 4 years ago. The supplementary nerve sonography showed some caliber irregularities, but there was a regular presentation of the roots C5 on both sides and C6 left side, so additional diagnostics were not clearly supportive for GBS.

For further clarification of the cytoalbumin dissociation, cerebrospinal fluid was taken from the ventriculoperitoneal shunt 3 days after the lumbar puncture. The findings were unremarkable (clear, protein 70 mg/dL, albumin 421 mg/L, 1 leucocyte/µL, polymorphonuclear, lactate 2.2 mmol/L, glucose normal 95 mg/dL, more details see Table 1) so that the diagnosis of GBS was discarded and intravenous immunoglobulins stopped. Virus PCRs (HSV-1-DNA, HSV-2-DNA, VZV-DNA, CMV-DNA, EBV-DNA, HHV-6-DNA) and a microbiological work-up were all negative, excluding common infections. In the course of further treatment with extended antibiotic therapy (meropenem) and intravenous admission of cortisone, adapted to the increased consumption during infection, the patient improved markedly. After appropriate rehabilitation measures, the patient regained his previous condition, also with regard to the tetraparesis. We, therefore, assumed an infection-associated encephalopathy in the context of pre-existing bifrontal infarcts and consecutive clinical mutism.

## 3. Discussion and Conclusions

Our case report demonstrates the difficulty of differential diagnosis in the case of a solely elevated CSF protein in a multimorbid patient. The comparative analysis of CSF derived from the ventriculoperitoneal shunt versus lumbar puncture could clarify the CSF findings in our case and confirm FS due to spinal stenosis.

In general, pathological causes of an elevated CSF protein fraction may be infections (bacterial, viral, or fungal), neuroimmunological diseases such as GBS, malignancies, intracranial hemorrhage, endocrinological diseases like hypothyroidism [2], degenerative disorders like Krabbe disease [3], metabolic disorders, intoxication or epilepsy [4]. However, a “moderately” increased CSF protein fraction can also occur within age-dependent normal ranges. Age-dependent normal values are available both for children [5] and adults [6,7], although adaptation to age is rarely performed during the clinical routine [8]. In our case, GBS was taken into consideration due to the clinical presentation, although CSF protein levels highly exceeded normal values. However, we could rule out GBS by additional CSF analysis, which was possible due to the pre-existing ventriculoperitoneal shunt. Alternative explanations like central nervous system infections or endocrinological causes were additionally ruled out. The slightly increased total protein fraction in the ventricular CSF was interpreted in the context of the patient’s age. However, differences in cell count and protein between ventricular and lumbar-derived CSF have been described (e.g., in meningitis; [9]); the exact dynamics and values when comparing CSF derived from ventriculoperitoneal shunts versus lumbar puncture still remain unclear.

**Figure 2:**
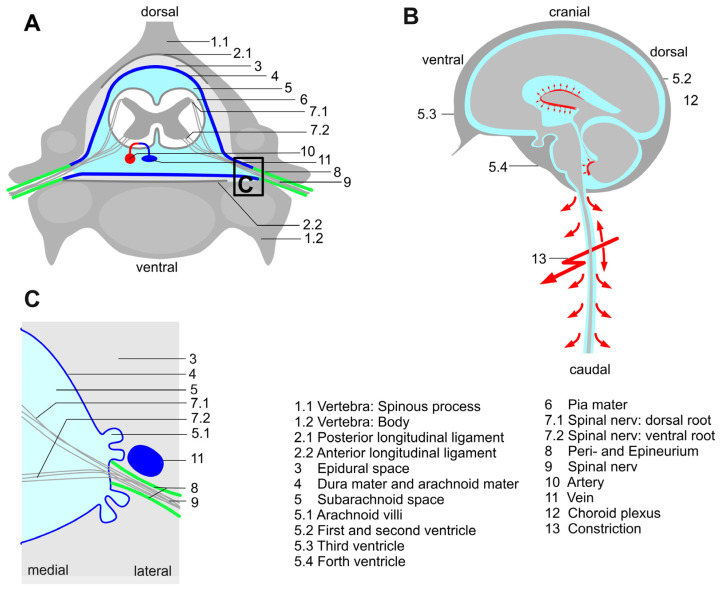
(**A**) Axial representation of spinal cord anatomical structures involved in cerebrospinal fluid (CSF) circulation. (**B**) Graphical presentation of CSF circulation with an impeded CSF circulation. (**C**) Mechanism of CSF drainage through arachnoid villi; It is hypothesized that through an impaired CSF circulation, the pressure in the CSF space below the lesion may be significantly reduced. As a result, the absorption of cerebrospinal fluid by the arachnoid villi might be impeded, leading to an altered composition of the cerebrospinal fluid.

FS was first described by Georges Froin (1874–1932) in 1903 as a triad of high protein content, xanthochromia, and marked coagulation [1,10] and associated with a spinal epidural abscess [11,12,13,14], tumors of various etiologies [15,16,17,18,19], spinal injury [20,21] or spinal stenosis as in the present case. All these pathologies go along with the compression of the CSF space within the spinal canal (Figure 2A–C). At the beginning of the 20th century, it was hypothesized that in Froin’s syndrome, a spinal obstruction interrupts all or part of the CSF circulation above and below the disorder [14,22,23,24]. The disturbed circulation leads to a characteristic mismatch between the CSF above the stenosis with regular findings and below with pathological findings [22,25]. As a possible explanation, the pressure in the CSF space below the lesion may be significantly reduced so that the absorption of cerebrospinal fluid by the arachnoid villi is impeded, leading to an altered composition of the CSF (Figure 2A–C, [23]). Recirculation of CSF below the stenosis into the CSF compartment above the stenosis may also be hampered in our case by the obstruction and possible pressure gradient despite the ventriculoperitoneal shunt. To date, there is only a single description (e.g., in a pediatric patient [19]) in which a comparative sample of the CSF was taken above the postulated stenosis via a ventriculoperitoneal shunt. As far as we know, this is the first published case that shows the different protein concentrations above and below spinal stenosis with the help of ventriculoperitoneal shunt and lumbar CSF sampling.

In conclusion, CSF protein elevations are a frequent finding, which can be attributed to various causes and are sometimes difficult to interpret in the clinical setting. Our case clearly demonstrates marked differences in the analysis of ventricular versus lumbar-derived CSF and highlights the complex mechanisms of CSF circulation. Further studies on the exact dynamics of both ventricular and lumbar-derived CSF, including pressure measurements, could contribute to a better pathophysiological understanding of CSF circulation.

## Figures and Tables

**Table 1:** Cerebrospinal fluid (CSF) findings from lumbar punction and puncture of the ventriculoperitoneal shunt.

	Lumbar Punction	Ventriculoperitoneal Shunt	Normal Findings
appearance	xanthochrome	clear	clear
erythrocytes	<1.000/µL	<1.000/µL	
nucleated cells	4/µL	1/µL	0–5/µL
leucocytes	4/µL	1/µL	0–5/µL
polymorphonuclear cells	25%	100%	
mononuclear cells	75%	0%	
lactate in the CSF	2.5 mmol/L	2.2 mmol/L	0–2.2 mmol/L
glucose in the CSF	50 mg/dL	95 mg/dL	
glucose in serum	66 mg/dL	137 mg/dL	
glucose CSF/serum ratio	0.8	0.7	>0.5
protein in CSF	938 mg/dL	70 mg/dL	0–45 mg/dL
albumin in the CSF	7240.0 mg/L	421.0 mg/L	
albumin in serum	24.9 g/L	25.3 g/L	34–48 g/L
albumin CSF/serum quotient	290.8 × 10^−3^	16.6 × 10^−3^	
IgG in CSF	1160.0 mg/L	60.1 mg/L	
IgG in serum	5.6 g/L	25.2 g/L *	7–16 g/L
IgG CSF/serum quotient	207.1 × 10^−3^	2.4 × 10^−3^	
IgG index	0.71	0.14	0.38–0.7

* note: CSF of the ventriculoperitoneal shunt was taken after the administration of intravenous immunoglobulins, so IgG in serum is not assessable.

## Data Availability

Data sharing is not applicable to this article as no datasets were generated or analyzed during this study.
